# Tree-Rings Mirror Management Legacy: Dramatic Response of Standard Oaks to Past Coppicing in Central Europe

**DOI:** 10.1371/journal.pone.0055770

**Published:** 2013-02-06

**Authors:** Jan Altman, Radim Hédl, Péter Szabó, Petr Mazůrek, Vladan Riedl, Jana Müllerová, Martin Kopecký, Jiří Doležal

**Affiliations:** 1 Institute of Botany of the Academy of Sciences of the Czech Republic,Průhonice, Czech Republic; 2 Faculty of Science, Department of Botany, University of South Bohemia, České Budějovice, Czech Republic; 3 Administration of Pálava Protected Landscape Area, Mikulov, Czech Republic; 4 Faculty of Science, Department of Botany, Charles University in Prague, Prague, Czech Republic; DOE Pacific Northwest National Laboratory, United States of America

## Abstract

**Background:**

Coppicing was one of the most important forest management systems in Europe documented in prehistory as well as in the Middle Ages. However, coppicing was gradually abandoned by the mid-20^th^ century, which has altered the ecosystem structure, diversity and function of coppice woods.

**Methodology/Principal Findings:**

Our aim was to disentangle factors shaping the historical growth dynamics of oak standards (i.e. mature trees growing through several coppice cycles) in a former coppice-with-standards in Central Europe. Specifically, we tried to detect historical coppicing events from tree-rings of oak standards, to link coppicing events with the recruitment of mature oaks, and to determine the effects of neighbouring trees on the stem increment of oak standards. Large peaks in radial growth found for the periods 1895–1899 and 1935–1939 matched with historical records of coppice harvests. After coppicing, the number of newly recruited oak standards markedly grew in comparison with the preceding or following periods. The last significant recruitment of oak standards was after the 1930s following the last regular coppicing event. The diameter increment of oak standards from 1953 to 2003 was negatively correlated with competition indices, suggesting that neighbouring trees (mainly resprouting coppiced *Tilia platyphyllos*) partly suppressed the growth of oak standards. Our results showed that improved light conditions following historical coppicing events caused significant increase in pulses of radial growth and most probably maintained oak recruitment.

**Conclusions/Significance:**

Our historical perspective carries important implications for oak management in Central Europe and elsewhere. Relatively intense cutting creating open canopy woodlands, either as in the coppicing system or in the form of selective cutting, is needed to achieve significant radial growth in mature oaks. It is also critical for the successful regeneration and long-term maintenance of oak populations.

## Introduction

Knowledge about the long-term development of forests is essential both for a theoretical understanding of present composition and structure, and for practical issues of management and conservation of forest ecosystems. Tree-rings offer an excellent opportunity to investigate forest history; dendroecological methods provide high spatial and temporal resolution [1]. Over the past few decades, dendroecology has become common in studying disturbances in semi-natural forests and a wide range of methods has been developed for identifying such disturbances using tree-ring data. All of these methods are based on the fact that trees which experience improved light conditions after the falling of neighbouring trees react with an abrupt increase in radial growth. This process is called *release*. Release events are inferred from tree-ring series if growth exceeds a given threshold. A review by Rubino & McCarthy [2] explored dendroecological methods used for the detection of historical disturbance events. Since then, several limitations of these methods have been discovered and additional criteria have been developed [3], [4].

**Figure 1:**
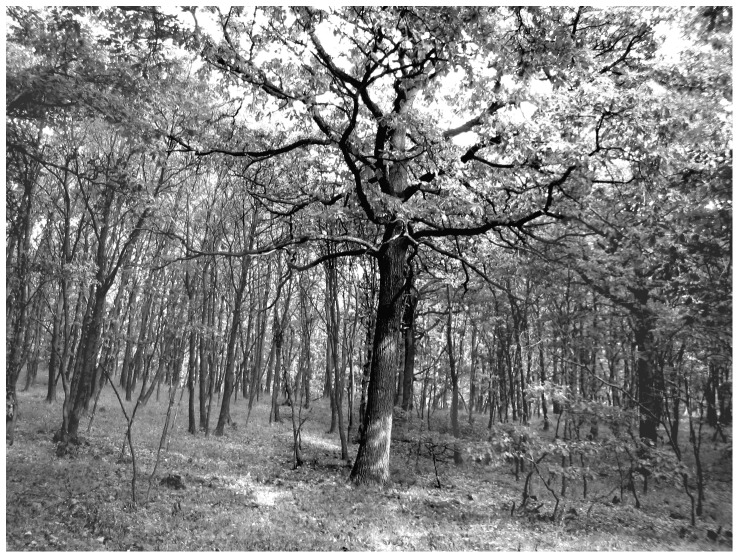
Typical oak standard with a large straight stem and spreading branches in an abandoned coppice-with-standards. Photo by R. Hédl.

Tree-ring research frequently focuses on remnants of natural forests. Such studies typically identify canopy disturbances following windbreak [5] or extreme climatic events, such as drought [6]. Forest management is an equally important factor responsible for changes in tree-rings [7]. Coniferous and broadleaved tree species positively respond to thinning or logging, which was demonstrated in several papers from North America [8]–[10]. Although the effects of recent canopy cutting on stem increment have often been studied, inferences about past management based on tree-rings are comparatively rare [11]. In Europe, various forms of woodland management have been practised for many centuries. The two most important management forms were coppicing and wood-pasture [12]. Coppicing can be traced in dendroarchaeological sources in prehistory [13] as well as in the Middle Ages [14]. Coppicing consisted of cutting trees close to the ground, letting them resprout from the cambium or dormant buds, and cutting the shoots repeatedly at short intervals. Combined with the coppice underwood, trees of generative origin (so-called standards) formed a characteristic feature of most coppices (Figure 1). In coppices-with-standards, the long-lived standards formed a scattered canopy over the short-rotation underwood. The density of standards was highly variable between sites and periods [15], [16]. Standards were usually oaks, but other species also occurred [16]. Coppice woods experienced periodic shifts of insolation. A coppicing event was followed by a few years of increased solar radiation to soil surface [17]. Soil warmed up faster, microbial activity was higher, litter decomposed quicker and nutrients were available in larger amounts. Periodically improved light and nutrient conditions are supposed to have had an immediate effect on the growth of standard trees, which can be detected as abnormally increased increment in their annual rings [18].

Coppicing was gradually abandoned in North-Western and Central Europe by the mid-20^th^ century, and the remaining few sites were retained for conservation purposes [18], [19]. Reports about the last regular coppicing are usually from the second half of the 20^th^ century [20], [21]. The abandonment of coppicing has had a significant impact on forest biodiversity [22]–[27]. Succession from open-canopy forests and forests with frequent alterations of light and dark phases (typical for coppicing) resulted in the decline of entire herb layer communities and species [25] as well as critically endangered invertebrates [28]. The abandonment of coppicing has had a negative effect on some tree species as well. Oaks have gradually lost their ability to successfully regenerate in many European woodlands. This phenomenon is often referred to as ‘oak decline’ [29]–[31]. Available studies attribute it to various factors [32], [33], e.g. plant diseases (e.g. *Microsphaera alphitoides*, *Phytophthora species*). Light availability is of particular importance in oak regeneration [34]–[37]. Due to low cutting intensity and subsequent competitive exclusion by shade-tolerant species [38], [39] oak is becoming unable to reach the mature phase. The frequent recurrence of light pulses in coppices may have conditioned the successful regeneration of oak and may have thus influenced the long-term survival of this species. In active coppice woods, light phases are followed by periods of increased competition for light. Throughout their lifetime, long-lived oak standards experience several cycles of light and dark phases, which is reflected in tree-ring increment. Information gained from tree-ring data can be compared to archival forestry management documents. Such research provides a fine example of the combination of the methods of ecology and history for solving common research questions [40]. Knowledge about the legacy of historical management in coppices-with-standards can contribute to detecting the causes of oak decline in European woodlands.

There exist only a handful studies dealing with the effects of coppicing on tree-ring release in oak standards. Jones [41] quoted Bartet [42], who noticed significant release right after coppicing. However, in only one out of six English woods [18], [43], [44] did standards show a clear release following coppicing. None of the four coppicing events after the 1940s was responsible for anomalies in tree-ring growth in the Bradfield Woods in Sussex, and at only one of four sites on the western fringes of London (Mad Bess Wood) did trees demonstrate release due to recent coppicing. In the Bradfield Woods, there was great variation in tree-ring growth between the cored standards, thus no evidence for the effect of coppicing on standards could be provided. This scarcity of information about tree growth dynamics in coppices-with-standards sharply contrasts with the relatively abundant knowledge on forest dynamics based on tree-rings in other forest types.

In this paper, we focused on detecting the effects of historical coppicing on the growth dynamics of oak standards in a former coppice-with-standards in Central Europe. Our aims were 1) to relate the releases detected in tree-rings of oak standards to historical coppicing events, 2) to assess the intensity of competition of neighbouring trees with oak standards, and 3) to search for possible connections between oak regeneration patterns and historical coppicing events.

## Methods

### Study site

**Figure 2:**
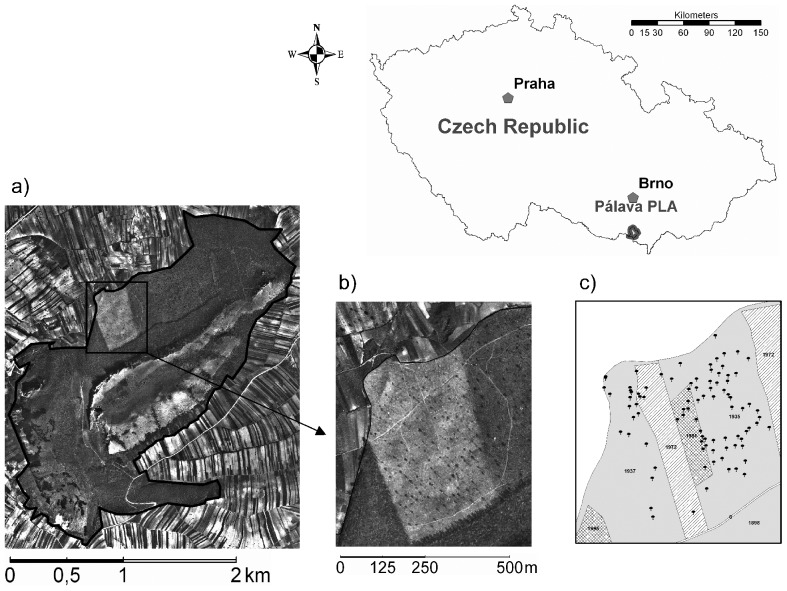
Location of the study site. The aerial photograph of Děvín Wood from 1938 (Military Geographical and Hydrometerological Office, Dobruška) depicts the last coppicing (a). The sample area (b) is visible as lighter rectangle with darker dots representing oak standards. c) shows the cored oak standards. The date of the last harvest is marked in each forest compartment.

The study area is located in Děvín Wood (48°52′N, 16°39′E), in the Pálava Protected Landscape Area, Czech Republic (Figure 2). Pálava is an ancient cultural landscape situated in the north-western edge of the Pannonian Basin with relatively warm and dry subcontinental climate. Děvín forms a conspicuous limestone crest in a gently undulating landscape, with altitudes ranging from 260 to 549 m a.s.l. Soil types are mainly weakly eluviated luvisols and leptosols (rendzinas) rich in carbonates with topsoil pH (water) from ca. 6 to 8, and fertile mull humus forms [45]. Děvín covers 381 ha, of which 262 ha are wooded mainly by thermo- to mesophilous oak-hornbeam woodland (*Carpinion*).

Děvín Wood was intensively managed for centuries. The oldest written evidence about traditional management is from the 14^th^ century and describes coppicing [15]. The cutting cycle gradually lengthened from the medieval 7 years to more than 30 years in the 19^th^ century. Scattered among coppice stools, there was a historically fluctuating density of standard trees, mainly oaks [15]. In the second half of the 20^th^ century, coppicing was abandoned in favour of high-forests and non-intervention conservation management. In 1946, a nature reserve was established at the site. Attempts to transform coppices to high-forests have lead to the singling-out of many coppice stools. Singling-out means selecting the strongest adult shoot on each stool and cutting the rest of the stems, thus forming a pseudo-high-forest. Stools were singled out several times in the second half of the 20^th^ century, e.g. in the 1970s and the 1990s. Today, the forest consists of high-forests, singled-out coppices with occasional old stools, and overgrown coppices. In many parts of the Wood the original standards are still clearly recognizable by their straight stems and characteristic branch architecture (Figure 1).

### Sample area

The coring of oak standards took place in the area of the last regular coppicing in Děvín Wood. The coppiced part (24 ha) is apparent on an aerial photograph from 1938 as a lighter rectangle with standards as darker dots (Figure 2). It includes the adjacent parts of two forest compartments. Compartments had relatively stable boundaries throughout the centuries and usually coincided with the extent of historical coppicing events. In the central part of the sample area, there were two clear-cuts (1971 and 1990), removing most of the surviving standards. In addition to these systematic thinning events, occasional cutting occurred from the 1970s to the 1990s.

The only detailed historical record describing standards (rather than underwood) in Děvín Wood comes from 1808 (Moravský zemský archiv/Moravian Archives F72 inv. č. 3041). In this survey, standards were counted and recorded separately for each species and compartment. Standards in the sample area were composed of hornbeam (*Carpinus betulus*) (48%), oak (*Quercus petraea*) (29%), ash (*Fraxinus excelsior*) (12%) and lime (*Tilia platyphyllos*) (10%), with 48 standards per hectare. The density of standards in 1938 (determined by counting them in the freshly coppiced area on the 1938 aerial photograph) was much lower – only 10 standards per hectare, i.e. one fifth of the 1808 value. The density of standards in 2011 was 6 standards per hectare.

### Data collection and analysis

Ninety stems of *Quercus petraea* agg. standards were cored in the winters of 2009–2011 and their exact geographical positions were recorded using differential GPS Trimble PathFinder Pro XRS. They represented all oak standards surviving in the sample area. Less frequent species, mainly *Carpinus betulus*, were omitted. Coring was carried out at a height of 1.3 m above ground surface, using a steel borer (Mora, Sweden). All cores were dried, mounted, sanded, and inspected for injuries, reaction wood and other aberrant features. Rings were counted from pith to bark and their widths measured to the nearest 0.01 mm using the TimeTable measuring device and PAST4 software (http://www.sciem.com). Ring-sequences were cross-dated visually using the pattern of wide and narrow rings, and verified using the program PAST4. A mean annual tree-ring width chronology was constructed and periods of oak establishment were determined on the basis of tree-rings.

To study neighbour effect on tree growth, all trees (3789 stems) with DBH >10 cm to a distance of 10 m around target oak standards were recorded. We opted for a distance of 10 m, because competition among trees is the most significant within this distance [46]–[49]. For each trunk we recorded the species, health status (living or dead), stem structure (multi-stemmed or single-stemmed) and measured the basal area of individual coppice stools.

### Historical analysis of forest management

The recent history of coppicing in the study area was established based on archival sources. Seven sets of forest management plans (FMP) from 1883 to 1971 were used with their corresponding maps although unfortunately not all maps survived (Moravský zemský archiv/Moravian Archives F72 inv. č. 3044, kniha 113 and 116, F121 kniha 5 and 31 and Ústav pro hospodářskou úpravu lesů/Forest Management Institute, Brno, Czech Republic arch. č. 16/34). The FMPs recorded the age of each compartment, from which the date of coppicing events could be calculated. The maps were georeferenced, vectorized, and completed with information from the FMPs using ArcGIS software. It is to be noted that extracting dates of coppicing from FMPs is not entirely straightforward: coppicing was done in the autumn/winter season, but FMPs were connected to calendar years. In addition, it is known that some parts of stands were sometimes cut in two consecutive years, but we do not know the spatial details. Possible distortions were taken into consideration in the analyses by employing a 3-year tolerance margin.

### Detection of release events

Release events were determined by *boundary line criteria* developed by Black & Abrams [3], who improved the formula of Nowacki & Abrams [11]. This method computes the percentage growth change (*%GC*) between average radial growth over the preceding 10-year period, *M_1_* (including the target year), and average radial growth over the subsequent 10-year period, *M_2_* (excluding the target year): *%GC* = [(*M_2_−M_1_*)/*M_1_*] * 100. After this, the prior growth for each tree-ring (a mean of radial growth over the 10-year period before the target year) was calculated. The boundary line was constructed by dividing prior growth data into 0.5 mm segments and the top ten values of %GC were averaged within each segment. Finally, linear, power, logarithmic, and exponential curves were fitted to all positive segment averages and the function with the highest *R^2^* was selected. The resulting equation determines the boundary line. Growth change values between 20% and 49.9% of the boundary line were identified as moderate releases and those between 50% and 100% as major releases [3].

The boundary line was calculated using datasets from 12 sessile oak chronologies from the ITRDB (International Tree Ring Database) [50] and one chronology from the study of Dolezal et al. [29] (Table S1). Calculations were done in the programme R [51] by the script for release detection [52] and the Dendrochronology Program Library (dplR) [53]. The dates of coppicing events extracted from FMPs were compared to the releases detected by the tree-ring analyses.

### Post-coppicing comparison of size and radial growth patterns between differently aged oak standards

To assess the growth pattern of oak standards in the past 50 years (after coppicing was abandoned in Děvín Wood), we compared the relative growth rate (RGR), cumulative stem diameter growth curves and resulting stem diameter structure for three groups of oak standards established before 1886, between 1886–1930, and after 1930. We also tested whether younger oak standards grew disproportionately more than older trees by relating the growth interval common for all trees for which information on growth history (tree-ring increments) was available (1953–2003) to prior stem size and age (1952). Finally, we assessed whether the three groups of trees that regenerated in different time periods preceding or following known coppicing events differ in various measures of crowding intensity from regenerating neighbouring trees (see next subchapter). Statistical differences in mean values of selected parameters were tested by analysis of variance.

### Analysis of neighbour effect on tree growth

Whether trees in the close vicinity of oak standards had any effect on the radial growth increment of the standards was evaluated by relating both diameter increment (DI, linear one-dimensional measure) and basal area increment (BAI) to several indices of local competition (CI, or index of crowding intensity) using linear regression. As the response variables, DI and BAI, were highly correlated with each other (r = 0.98), we present only results using the first measure of radial growth. To assess the influence of neighbours on target tree growth (relative growth rates calculated as RGR = [ln (*y_i+1_*)−ln (*y_1_*)]/yr, where *y_i+1_* is final stem diameter, *y_1_* is initial diameter, and *yr* is the length of the growth period in years), several CIs were calculated for each tree for which information on growth history was available to account for size-related neighbour effects, and for intraspecific vs. interspecific interference [54]. CIs were calculated as (1) the sum of individual basal areas of all neighbours within a circle of 10 m radius around the target stem, (2) the sum of stem basal areas of individual species, and (3) the sum of individual species stem basal areas divided into distinct categories based on health status (living or dead) and stem structure (multi-stemmed or single-stemmed, the latter representing singled-out coppice stools). Given the relatively low density of standard trees and because we do not assume any significant regeneration from seeds, most, if not all, neighbouring trees have resprouted from coppice stools. The neighbour effect of each CI was first analysed by a univariate regression model and analysis of variance to compare the strength of competition between the three groups of trees that regenerated in different time periods preceding or following known coppicing events.

Furthermore, to take into account the possible interaction between individual predictors, we modelled the neighbourhood effects of all CI indices using conditional inference trees (CIT, a type of classification and regression tree). This method belongs to non-parametric regressions that display a binary tree built by a process of recursive partitioning. CIT have been shown to give results that are comparable to those of traditional regression trees, but without their failings (overfitting and a biased selection of covariates when forming splits) [54]. CIT use a permutation-based statistical framework to ensure an even-handed selection of covariates and to stop splits being formed if they are not significant at some pre-specified level of significance (we used the 5% level of significance). The P-values were adjusted for multiple testing using the Bonferroni correction. The analysis was performed with the Party 1.0-3 package [55] in the R 2.13.1 program [51].

To evaluate possible temporal changes in competitive interactions during stand development following the last coppicing events, we included dead trees (visible as stump remnants) into the neighbourhood analyses. Such trees can play a significant role in competition in the early stages of stand development following coppicing. By analyzing simultaneously the effects of dead and living trees, we tested the prediction that a temporal shift took place in the mode of interaction from severe competition in the early phases of stand development resulting in tree mortality to more or less stable conditions following stand self-thinning. The neighbourhood analysis was conducted for a longer growth interval covering the entire post-cutting period 1953–2003, during which most oak standards showed a decline in radial growth resulting from decreasing light availability. To assess whether neighbour effect on tree growth changed over this period, we conducted complementary analyses for the separate intervals of 1953–1972 and 1973–2003.

## Results

### Coppicing and radial growth of oak standards

The average age of oak standards was 106 years and ranged from 28 years to 146 years. The first boundary line for *Quercus petraea* was computed on the basis of 45,755 tree-rings from 366 trees. The boundary line was fitted by an exponential function with the equation *y = 5.0067 e^−0.664x^*, which had the highest *R^2^* of 0.93. We identified altogether 126 releases (35 moderate, 91 major) across all tree-rings. The average number of release events per tree was 1.4.

**Figure 3:**
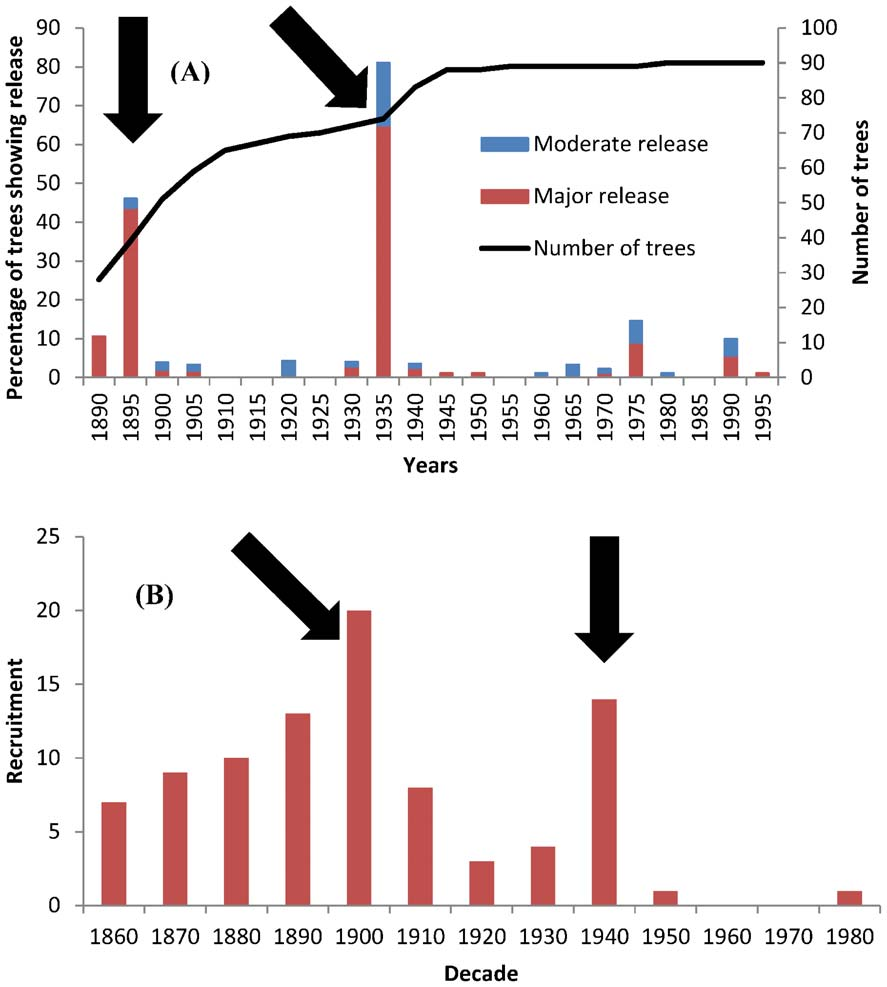
(A) Percentage of trees showing release in 5-year intervals, as identified with the boundary-line release criteria. The two main releases closely followed coppicing events. Releases in the 1970s and 1990s coincide with the major singling-out of coppice stools. (B) Number of trees established in individual decades (age was determined on the basis of increment cores taken at breast height). With two exceptions, all oak standards originated before or shortly after the last regular coppicing in 1935/1937. The two main coppicing events are indicated by black arrows.

**Figure 4:**
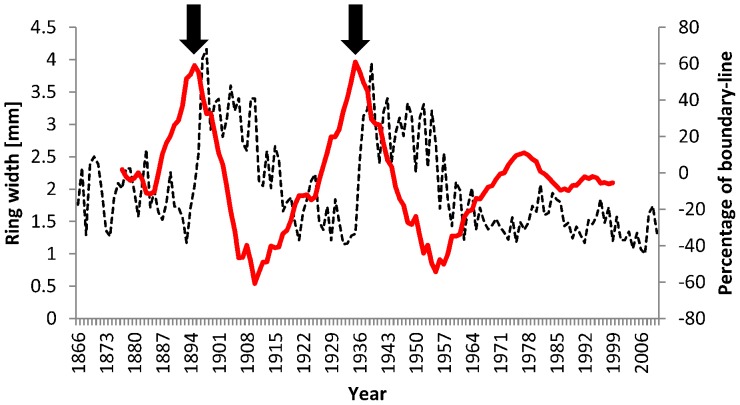
The average annual radial growth of *Q. petraea* standards (dashed line) and values of boundary-line for this mean growth (red line). The two main coppicing events are indicated by black arrows.

**Figure 5:**
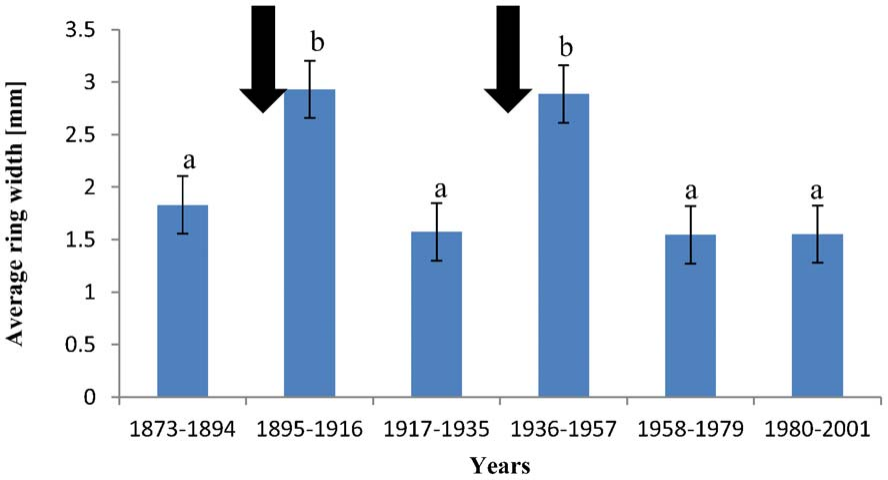
Average tree-ring growth for 22-year time periods. Arrows mark the last two historical coppicing events. The periods after coppicing had significantly higher average tree-ring increments than the periods not following coppice events. Columns sharing the same letter are not significantly different at *p<0.01* (ANOVA followed by Tukey's HSD post-hoc tests). Error bars represent standard error. The period 1917–1935 includes a 19-year time span, because there are no more years before the first coppicing event (1935).

A disturbance chronology was constructed for the period from 1890 to 1999 for 5-year segments. Releases occurred with four exceptions in all 22 segments and the percentage of trees showing release varied substantially. However, large peaks in disturbance events were detected for the periods 1895–1899 and 1935–1939 (Figure 3a). A higher proportion of disturbance events was identified also in the second half of 1970s and the first half of 1990s, but these events were not as pronounced as the previous ones (Figure 3a). Mean annual tree-ring width chronology showed highest values in the years 1897 and 1939 after abrupt increases in growth (Figure 4). The boundary line method detected two major releases for the mean chronology in the years 1895 and 1935 (Figure 4). We observed abnormally high average tree-ring growth for a 22-year period following both coppicing events. These periods were statistically significantly different from other periods (Tukey's HSD Post-hoc test, P<0.01, Figure 5).

Releases detected by tree-ring analysis were related to historical records of coppice events. According to the FMPs, the analysed compartments were coppiced in 1895–1897 and in 1935–1937. Parts of the sample area were felled in the 1970s (25% of the area) and the 1990s (6%), related to attempts to transform the coppice-with-standards to high-forest. Twenty-six trees experienced both historical coppicing events. Over 90% of the detected releases could be matched with historical coppicing events within a 3-year tolerance limit, which was introduced because of the inaccuracies inherent archival sources. Further releases were detected in the second half of the 1970s and the first half of the 1990s when coppice management already ceased. The number of these releases was relatively small compared to those following coppicing events. Unfortunately it was not possible to locate these recent events precisely in space and match them with individual trees, because cutting was scattered.

### Comparison of size and radial growth parameters between differently aged oak standards after the abandonment of coppicing

**Figure 6:**
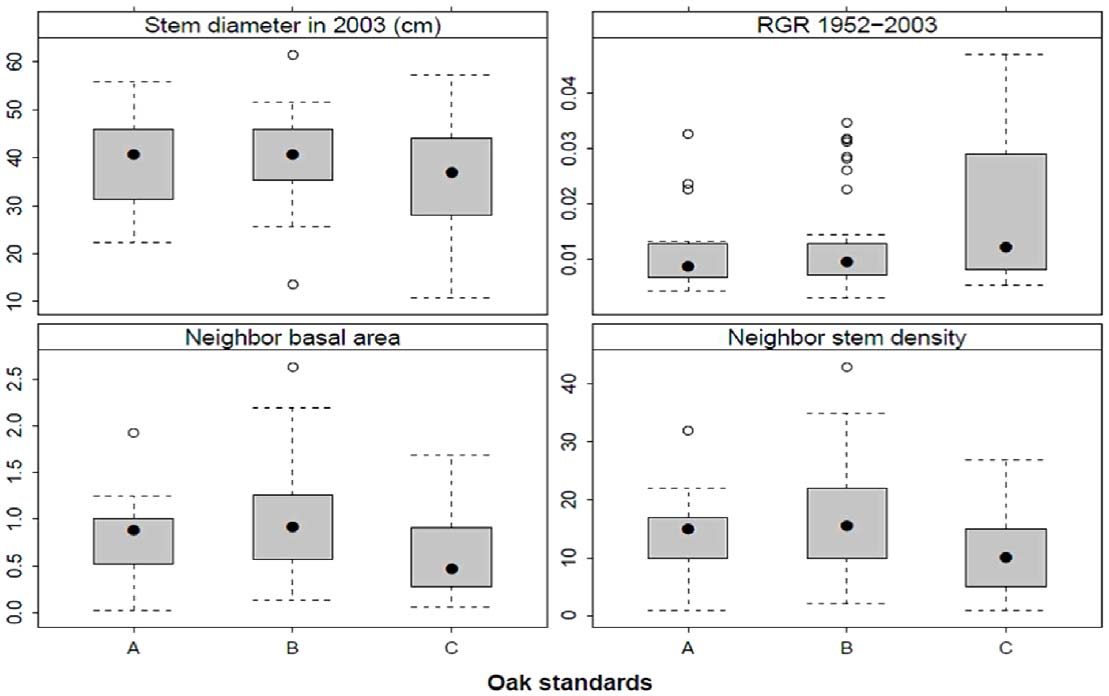
Comparison of stem diameter, relative growth rate (RGR) and competition indices (sum of basal area of all living and dead neighbours, and the density of dead neighbouring stems) of oaks established before 1886 (A), between 1886 and 1930 (B), and after 1930 (C). Boxes represent 25–75% of values, black dots medians, whiskers 1.5 interquartile ranges, and open dots outliers.

**Figure 7:**
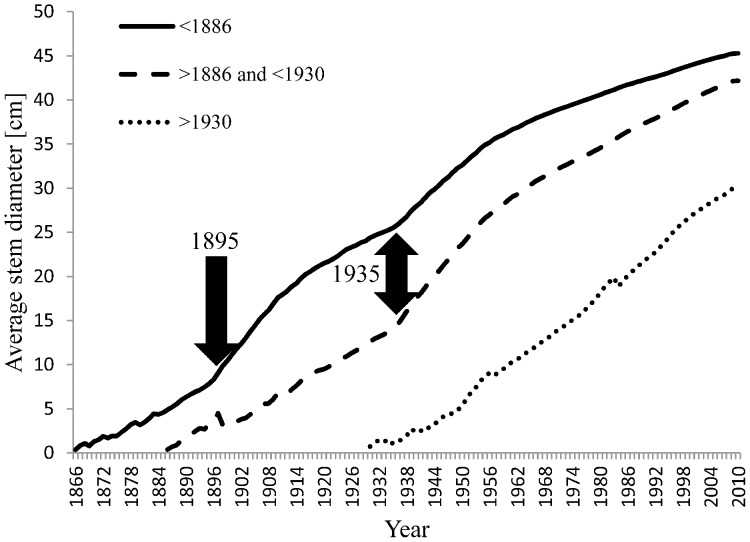
Mean cumulative stem diameter growth curves for three groups of oak standards established before 1886, between 1886 and 1930, and after 1930 with respect to the two coppicing events (marked by thick arrows).

**Figure 8:**
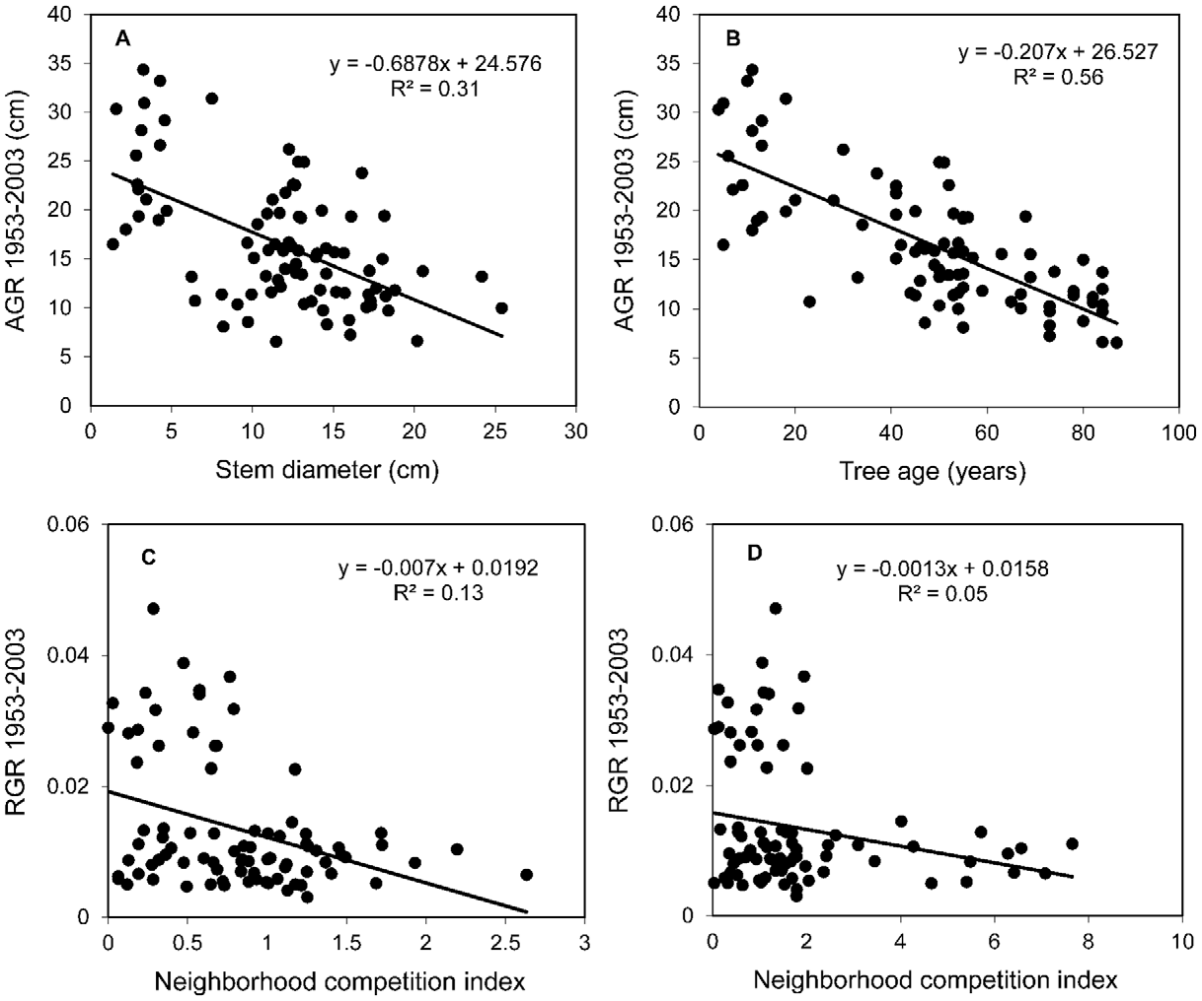
Relationships between: (a) diameter increment from 1953 to 2003 (AGR, absolute growth rate) and stem diameter in 1952; (b) AGR and tree age in 1952; (c, d) relative growth rate (RGR) of diameter increment and the intensity of neighbourhood competition (crowding) within 10 m; where in (c) basal areas (in m^2^) of all dead trees were considered, while in (d) *Tilia platyphyllos* living and dead trees were analysed.

When oak standards established before 1886, in the period 1886–1930 and after 1930 were compared in terms of radial growth, size parameters and cumulative increment curves, those established after 1930 had significantly higher RGR in the period 1952–2003 (ANOVA, P = 0.023, Figure 6), and were still growing actively with no sign of growth decline (no apparent asymptote) (Figure 7). Despite higher relative increments, trees established after 1930 still had a significantly smaller stem diameter in 2003 (P = 0.012, Figure 6) than asymptotically-growing older oak standards that showed a decline in radial growth after 1950. Size- and age-growth regressions (Figure 8 a, b) revealed that the diameter increment of oak standards from 1953 to 2003 was negatively correlated with stem diameter and tree age in 1952, indicating that younger oak standards grew disproportionately more than older conspecifics. This is likely to have been caused by recently reduced competition. Neighbouring stems around younger standards are significantly less dense and have lower basal area (P<0.05, Figure 6).

### Neighbour effect on the growth of oak standards

On average 43.5 stems (31% dead and 69% living) were recorded around target oak standards to a distance of 10 m. The majority of neighbouring stems (86%) had a multi-stemmed structure, which proves that they had originated from coppice stools. The most abundant species in the vicinity of oak standards was *Tilia platyphyllos* (2435 stems) making up 64% of the total number of neighbours (1501 living stems and 851 dead stems were coppice shoots, and 83 stems were living single-stemmed trees), followed by *Carpinus betulus* (572 stems, of which 259 were coppice shoots), conspecific oaks (356 stems, mostly living) and *Fraxinus excelsior* (231 stems, mostly living). The remaining 15% were *Acer campestre*, *Acer pseudoplatanus*, *Acer platanoides*, *Ulmus minor*, *Populus tremula*, *Cerasus avium*, *Sorbus torminalis*, *Cornus mas*, *Betula pendula* and *Castanea sativa*. Among these, only *Acer campestre* had more than one percent (60 stems) of the total number of neighbouring stems.

The competition indices that were significantly negatively correlated with the diameter increments incorporated mainly interspecific effects of *Tilia platyphyllos* living and dead trees (Figure 8 c, d). The growth reductions of oak standards due to competition from other tree species were non-significant. Explained variance (adjusted *r^2^*) from the significant univariate regressions of RGR of oak from 1953 to 2003 on those competition indices ranged from 4.3% to 13.7%, and it increased mostly when dead *Tilia* individuals were used to define a local neighbourhood. In fact, the single competition index that accounted for the most variation was based solely on basal areas of multi-stemmed *Tilia* trees (adjusted *r^2^* = 0.073, *P* = 0.02). The separate analyses for the two periods 1953–1972 and 1973–2003 revealed a stronger competitive effect of neighbouring trees for the first period 1953–1972, when *Tilia* neighbours explained 13.5% variability in radial growth increments compared to 9.3% variability explained in the second period. Permutation-based conditional inference trees (CIT) supported the results of univariate regressions, showing primarily the effect of dead and living *Tilia platyphyllos* trees on the growth reduction of oak standards.

### Relationship between coppicing and oak regeneration

The rate of recruitment of trees established between 1860 and 1899 (64% of cored oaks) was relatively even, as opposed to those established in the 20^th^ century (Figure 3b). After coppicing events, the number of trees that survived from establishment grew markedly. Most of the surviving trees were from the decades following coppice events - 20 trees (22% of cored oaks) established during the decade after the 1896–7 coppicing event and 14 trees (16%) after the 1935–7 coppicing event have survived until the present, while for other decades the average is 5 trees (Figure 3b).

## Discussion

### Effects of coppicing on the growth of oak standards

In Děvín Wood, the periods of enhanced radial growth of standard trees started with pronounced peaks and lasted for about two decades before levelling to normal values. We were able to match these events with the dates of known coppicing events, hence providing unique evidence for past coppicing in the radial growth of standard trees. Percentage change in radial growth allowed us to distinguish severe (major release) disturbance events from mild (moderate release) events [3]. We identified a much higher proportion of major releases than of moderate ones due to coppicing. From an ecological perspective, coppicing at our study site can therefore be considered as a severe disturbance event. Dendrochronological methods used for disturbance analysis are based on the change of light availability for surviving trees after disturbance events [3], [11], [56]. Light pulses following coppicing were probably the main factor inducing abrupt growth changes in standards.

### Explaining the effects of coppicing

It seems that the increase in radial growth in standard oaks after coppicing is controlled by the intensity of competition with underwood. This competition is temporarily abolished or lowered after coppicing, enabling standards to grow beyond their usual increment. This effect gradually declines as the resprouting underwood regains the space between standards. The period of increased growth after coppicing in Děvín Wood was much more longer (22 years) than the period documented in other studies, which ranged from 6 years [57] through 7–12 years [58] to 15 years [59]. The site-specific setting of environmental conditions, stem density, species composition and the size of the gaps may lead to contrasting patterns [11], which probably applies to the reaction of standards to coppicing as well. Competition for light is negligible when the underwood is too small to compete for light with the protruding standards. However, as the coppicing cycle lengthened to several decades, competition for light could have had a significant effect. The same is true for young standards before they reach the canopy and are not shaded anymore. Nevertheless, there can also be other factors influencing radial growth.

The increased availability of soil nutrients after removing the dense underwood (usually in winter months) can be important at nutrient-poor sites. According to this view, increased solar radiation in early spring enhances soil microbial activity [18], mobilizing soil nitrogen that can be utilized by standards to jump-increase their radial growth. However, in Děvín Wood the substrate is exceptionally fertile. Soils are deep loamy-clayey slope accumulations with a high pH (6–8 in water) and a mull type of humus indicating very fast litter decomposition. These soils are rich in organic matter, nitrogen and base cations, providing sufficient nutrients [45].

Another factor responsible for coppicing-related releases in Děvín Wood could be competition for water. The site has relatively warm and dry climate and soil water is deficient. In the vegetation season from April to September, precipitation is only 367 mm and average temperature 16.1°C (Mikulov and Perná, data for 1947–1978, Czech Hydrometeorological Institute). Periods of drought occur frequently posing the most important constraint for plant growth. Consequently, temporary reduction of competition for water might have triggered the abnormal tree-ring increments following coppicing events in Děvín Wood. By contrast, no soil water limitations may have caused the failure to detect coppicing at English sites [18], [43].

### Growth of standards is influenced by neighbouring trees

We found that the species composition of competing underwood can be an important factor for the growth of standards. Over 95% of *Tilia* in Děvín Wood is *T. platyphyllos* [60]. It resprouts from coppice stools in large numbers at the site. A typical multi-stemmed stool of *Tilia* has ca. 10, sometimes up to 20 shoots (H. Malíková, unpublished data). Because the oak standards were taller than resprouting *Tilia*, competition for light is not the sole explanation for the effect *Tilia* had on the growth of standards. We suggest that this effect was also driven by competition for soil resources because *T. platyphyllos* develops very dense roots in the topsoil in order to satisfy its exceptionally high demands for water, and sustain its relatively high concentration of nutrients in leaves [61], [62]. Dense coppice shoots of *T. platyphyllos* were a major competitor for light and soil resources to the surrounding trees including oak standards in the early stages of stand development following coppicing. The neighbourhood analyses demonstrated the negative effects of neighbours (mainly former underwood trees) on stem diameter increment of oak standards in 1953–2003. The neighbourhood model fitted best when the neighbouring trees of *Tilia* were included, while other tree species including conspecific neighbours had a minor impact. This supports the assumption of non-equivalent neighbour effects [63]. The strongest growth reduction in oak standards was explained by dead *Tilia* trees, i.e. remnants of coppice stools included in the mapping of neighbouring trees in 2012. These *Tilia* trees were probably established after the last coppicing and perished gradually through competition. This process slowly improved growth conditions for oak standards. Standards exhibited a stronger negative effect during the first 20–40 years following the last major coppicing event in the 1930s than in later periods.

### Oak decline and management history

Information about newly established oak standards completes our knowledge about the performance of oak standards. Standard dendrochronological methods cannot reconstruct the processes of the early stages of tree development. On the basis of our results, we cannot directly infer the reasons for the oak decline [29], [30], because our analysis involved only relatively old oak individuals. However, the establishment of new oak standards clearly followed the coppicing events. The analysis of oak recruitment in Děvín Wood revealed a significant increase in the number of trees established after both coppicing events, and virtually no recruitment in the decades after the 1940s, when the site became a reserve with restricted management. At other sites, this synchronous trend between tree recruitment and disturbance events was documented primarily after large natural disturbances [64]–[67] but also after harvesting [65], [68]. The analysis of 206 vegetation plots sampled in Děvín Wood in 2002–2003 [60] showed that oak seedlings occurred only rarely. Currently, oak does not regenerate in Děvín Wood at all. The last significant regeneration of oaks that subsequently reached the phase of mature trees had been in the 1940s, following the last regular coppicing.

Oak seedlings perform better under high insolation than other dominant tree species, such as beech [69], [70]. Oak cannot stand competition with shade-casting and shade-tolerant tree species in the long run [39]. Under unfavourable conditions, most oak seedlings grow for about five years and subsequently die if light conditions do not improve [71]. In forest environments, favourable conditions for successful oak regeneration include open canopy [37], [38] and non-shading understorey vegetation [72]. Although it is not clear how open forests could have been maintained in prehistory [73], coppice management was certainly capable of creating suitable conditions for oak regeneration in the past millennium [15], [16]. Human influence through management is therefore likely to have contributed to the long-term presence of oak in European woodlands.. In today's shady, closed-canopy European forests, opening up the canopy may be the only possible way for oaks to reach maturity.

## Supporting Information

Table S1 **Tree-ring data sources used in the development of boundary-line and absolute increase threshold (ITRDB = International Tree Ring Database).**
(DOCX)
