# Supplementary material for: Tree-Rings Mirror Management Legacy: Dramatic Response of Standard Oaks to Past Coppicing in Central Europe
Source: PLoS One. 2013 Feb 6;8(2):e55770. doi: 10.1371/journal.pone.0055770 (PMC3565998; doi:10.1371/journal.pone.0055770)
Supplement: Table S1 — Tree-ring data sources used in the development of boundary-line and absolute increase threshold (ITRDB = International Tree Ring Database). (DOCX) [file pone.0055770.s001.docx]

| Country | Site | Source | Contributor |
| --- | --- | --- | --- |
| Austria | Weinerwald | ITRDB | G. Strumia |
| Czech Republic | Certoryje | Dolezal et al. [30] | P. Mazurek |
| France | Foret de Chinon | ITRDB | J.R. Pilcher |
| Germany | Ebrach | ITRDB | B. Becker |
| Turkey | Buyukduz Forest | ITRDB | A. Petrucci, P.I. Kuniholm,  J.S.Terrell, L. Steele |
| United Kingdom | Maentwrog | ITRDB | M.K. Hughes |
| United Kingdom | Padley Wood | ITRDB | R. Morgan |
| United Kingdom | Bath | ITRDB | J.R. Pilcher |
| United Kingdom | Cappoquin | ITRDB | J.R. Pilcher |
| United Kingdom | Enniscorthy | ITRDB | J.R. Pilcher |
| United Kingdom | Killarney | ITRDB | J.R. Pilcher |
| United Kingdom | Lough Donn | ITRDB | J.R. Pilcher |
| United Kingdom | Rostrevor | ITRDB | J.R. Pilcher |
